# The impact of basic public services on residents’ consumption in China

**DOI:** 10.1057/s41599-022-01367-2

**Published:** 2022-10-25

**Authors:** Xing Xiong, Xinghou Yu, Yuxin Wang

**Affiliations:** 1 Research Center for Economy of Upper Reaches of the Yangtze River, Chongqing Technology and Business University, Chongqing, China; 2 School of Economics, Chongqing Technology and Business University, Chongqing, China

**Keywords:** Economics, Economics

## Abstract

China has the largest market potential in the world, but the resident consumption rate is relatively low. Releasing China’s consumption potential will contribute to the sustainable growth of China’s economy and the global economic recovery. Based on the supply of basic public services, this study analyzes the income and substitution effects of basic public services on residents’ consumption, with a view to finding channels and measures to stimulate residents’ consumption from the public sector. We first used the TOPSIS method of entropy weight to evaluate the public service level of 31 provinces in China, and then built a regression model to analyze the impact of public services on residents’ consumption, urban residents’ consumption, rural residents’ consumption, and the consumption gap between urban and rural residents. The study found that the improvement of public services has a significant positive effect on consumption, while public services also have a positive effect on narrowing the consumption gap between urban and rural residents, but the impact on urban and rural residents is different, and the positive effect on urban residents’ consumption is higher than that of rural residents. Based on the research results, this paper puts forward policy implications. On the one hand, we should increase the expenditure on basic public services, and on the other hand, we should optimize the layout of basic public services between urban and rural areas.

## Introduction

Since 1978, China’s economic aggregate has grown gradually and has become the second largest economy in the world. However, China has an obvious urban–rural dual economic structure, which is still in a state of insufficient domestic consumption. Investment, export, and consumption are the “troika” driving China’s economic growth, but in recent years, great changes have taken place in both the total volume and structure. In terms of investment, although investment has played a huge role in promoting China’s economic growth in the past, it is also facing the problem of diminishing marginal effects. In terms of exports, with the instability and uncertainty of the international environment, economic globalization and COVID-19 have seriously impacted China’s export trade. In terms of consumption, China’s household consumption has been sluggish for a long time, accounting for <40% of GDP in the past 15 years. According to the development experience of developed countries, when the per capita GDP exceeds 10,000 US dollars, the proportion of household consumption in GDP will generally exceed 50%. In 2019, the consumption rate of Chinese residents will be only 38.8%, which has huge room for improvement. Therefore, promoting consumption in an all-round way has become a necessary measure for China to cope with medium—and long-term challenges and maintain economic resilience.

As an important part of the government’s public expenditure, public services play a positive role in improving social welfare, while helping to release residents’ consumption potential and promote sustainable economic development. In the context of the global economic downturn, improving and strengthening public services will help increase people’s effective consumption and promote economic recovery. Keynesian contingent fiscal policy also emphasizes that when the total social demand is less than the total social supply, expansionary fiscal policy should be implemented to increase fiscal expenditure to increase effective social demand and promote economic development (Leah Platt Boustan, 2013). Since 1978, China’s economy has achieved rapid growth relying on investment and exports. However, in recent years, due to the global economic downturn, China’s exports have been affected, and overcapacity has led to a decline in investment. Therefore, raising the consumption level of Chinese residents is a realistic choice to promote China’s economic recovery.

However, the consumption rate of Chinese residents is at a relatively low level, which restricts the development of China’s economy. According to Chinese statistics^1^, since 1978, China’s economic development has contributed to the increase in personal income. From 1978 to 2019, the per capita disposable income of urban residents in China increased by about 123 times, and the per capita disposable income of rural residents increased by about 112 times. However, the growth rate of household consumption is far lower than that of income. The consumption level of urban residents only increased by 92 times, while that of rural residents increased by 108 times. According to the United Nations, the final consumption rate of Chinese residents in 2017 was 38.7%, while the United States, Britain, Germany and Japan reached 68.4%, 65.7%, 52.9%, and 55.5% respectively, in the same period^2^. In general, the consumption level of Chinese residents lags behind the income level and economic development level. The reason for the insufficient demand of Chinese consumers is the uncertainty of expectations for the future. Preventive savings theory emphasizes that the reason for high savings and low consumption is to deal with the uncertainty of preventive savings in the future (Peter, 2018). Due to various demands, residents choose to reduce the proportion of unnecessary consumption in current consumption expenditure and increase preventive savings. Therefore, China’s overall consumption is insufficient, and the consumption difference between urban and rural residents is large.

For a long time, China’s economic development has been unbalanced and inadequate. Compared with underdeveloped areas and rural areas, higher quality education resources, higher skilled health technicians, and better medical facilities are concentrated in developed areas and urban areas. This makes the inequality of residents’ opportunities caused by the difference in individual access to education resources, medical resources, and social security resources quite serious. Roemer’s (2002) equal opportunity theory divides the factors that affect economic individuals into “environment” and “effort” variables and believes that the effort inequality caused by controllable “effort” factors is reasonable and is caused by the difference in the availability of development resources. The opportunity inequality caused by uncontrollable external “environmental” factors is unreasonable. According to Romer’s equal opportunity theory and the fact that China’s economic development is unbalanced and insufficient, it can be found that the unequal opportunities caused by the differences in access to education resources, medical resources, social security, and other resources are unreasonable. Therefore, the supply of basic public services actually promotes equality of opportunity, enabling individuals to have equal access to educational resources, medical resources, and social security resources, thus reducing the degree of inequality of opportunity caused by “environmental” factors, thus promoting residents’ consumption. Establishing a sound social security system and high-quality public service supply will help reduce the release of preventive savings and consumption potential. Public service is an important means to stimulate Chinese residents’ consumption. Improving public services not only helps to improve the welfare level of Chinese residents but also helps to release the consumption potential of Chinese residents, thus promoting the sustainable growth of China’s economy.

This paper contributes to the literature in two ways. First of all, in developed economies, the proportion of public service supply and consumption is relatively high, while the impact of public services on residents’ consumption is relatively small. We added evidence from developing countries that have low public service levels and low consumption to problem ratios and analyzed their impact on Chinese residents’ public service consumption. Therefore, we have enriched the literature on these impacts in developing economies by providing evidence for improving residents’ welfare and economic development. Secondly, this study not only discusses the overall impact of public services on residents’ consumption but also analyzes the impact of public services on the consumption gap between urban and rural residents, providing ideas for solving the problem of urban-rural dual economic structure in China.

## Models and data

### Model and variable demarcation

We use ordinary least square (OLS method) to test the impact of public services on Chinese residents’ consumption. Due to the different resource endowments of urban and rural residents, the impact of public services on urban and rural residents’ consumption is also different. Therefore, on the basis of analyzing the overall impact of public services on Chinese residents’ consumption, we analyzed the different impacts of public services on urban residents’ consumption and rural residents’ consumption and analyzed the impact of public services on the consumption gap between urban and rural residents in China. Formula (1) is the overall impact of public services on Chinese residents’ consumption. Formula (2) is the impact of public services on the consumption of Chinese urban residents. Formula (3) is the impact of public services on rural residents’ consumption in China. Formula (4) is the impact of public services on the consumption gap between urban and rural residents in China.

1 $${\rm {HC}}_{it} = \alpha _0 + \alpha _1{\rm {PS}}_{it} + {\rm {CONTROL}}_{it} + \varepsilon _{it}$$

2 $${\rm {UC}}_{it} = \alpha _0 + \alpha _1{\rm {PS}}_{it} + {\rm {CONTROL}}_{it} + \varepsilon _{it}$$

3 $${\rm {RC}}_{it} = \alpha _0 + \alpha _1{\rm {PS}}_{it} + {\rm {CONTROL}}_{it} + \varepsilon _{it}$$

4 $${\rm {URG}}_{it} = \alpha _0 + \alpha _1{\rm {PS}}_{it} + {\rm {CONTROL}}_{it} + \varepsilon _{it}$$

HC_it_ represents residents’ consumption level, which is measured by residents’ consumption rate. UC_it_ refers to the consumption level of urban residents, which is measured by the consumption rate of urban residents in this paper. RC_it_ is the consumption level of rural residents, and we use the consumption rate of rural residents to measure it. URG_it_ refers to the consumption gap between urban and rural residents, and we use the ratio of consumption gap between urban and rural residents to measure it. PS_it_ refers to the public service level, based on the public service indicator system, the entropy weight TOPSIS method is used to calculate PS_it_. CONTROL_it_ are the control variables. Indicators of each variable are shown in Table 1.

**Table 1:** Variable Description.

Explained variable	Household consumption level (CR)		Household consumption rate (%)
	Consumption level of urban residents (URCR)		Consumption rate of urban residents (%)
	Consumption level of rural residents (RUCR)		Consumption rate of rural residents (%)
	The consumption gap between urban residents and rural residents (GAPCR)		The difference between the consumption rate of urban residents and rural residents (%)^a^
Explanatory variables	Public service (PS)	Public basic education	Student–staff ratio of primary (%)^a^
			Student–staff ratio of junior (%)^a^
			The government funds for education per student (yuan)
		Medical and health care	Per capita local government expenditure on medical and health care (yuan)
			Number of health technicians per 10,000 people (number of people)
			Number of beds in medical institutions per 10,000 people (unit)
		Social security and employment services	Per capita local government expenditure on social security and employment (yuan)
			Health insurance coverage (%)
			The registered urban unemployment rate (%)^a^
		Public welfare infrastructure	Per capita local government expenditure on transportation (yuan)
			Density of Highway (m/m^2^)
			Per capita number of public transport vehicles in operation (unit)
		Public ecological environment	Per capita local government expenditure on environmental protection (yuan)
			Harmless treatment rate of household waste(%)
			Green coverage rate in built-up areas(%)
		Public cultural services	Per capita local government expenditure on culture, sports and media (yuan)
			Per capita public library holdings (Copies/person)
			The proportion of cable broadcasting and television subscribers in the total number of households (%)
Control variables		Level of industrial structure (IS)	Output Value of Tertiary Industry/Output value of secondary industry
		Urbanization level (UR)	Urbanization rate (%)
		Level of economic development (PGDP)	Per Capita GDP(yuan)

^a^Denotes negative indicators, while others are positive indicators.

We designed the public service evaluation index system according to the theory of Ocampo et al. (2019) public service evaluation indicator system including six aspects: Public basic education, medical and health care, social security and employment services, Public welfare infrastructure, Public ecological environment, Public Cultural Services. The public service evaluation index system is shown in Table 1.

According to the public service evaluation index system, the entropy weight TOPSIS method is used to measure the public service level of 31 provinces in China. The entropy weight TOPSIS method is a combination of the TOPSIS method and entropy method. The main principle of the TOPSIS method (Olson, 2004) is to sort the evaluation objects according to the distance between the evaluation object and its ideal target. However, when the evaluation environment or its own conditions change, the optimal solution and the worst solution will change, which will lead to the inconsistency of the evaluation results. Therefore, on the basis of the TOPSIS method, the entropy method is used to determine the weight of each evaluation index, and the index system is weighted according to the numerical difference of the index value (Tao and Feng, 2018). In this way, the impact of variable factors on the evaluation results can be effectively eliminated, and the evaluation results are more comparable.

The calculation steps of entropy weight TOPSIS method are as follows.

Step 1: Same trend processing of variables.

Suppose that the decision problem has n evaluation schemes and m evaluation indexes, and the decision matrix composed of the original evaluation values is *X* = (*X*_*ij*_). Since there are positive and negative indicators in the evaluation indicator system, it is necessary to first conduct the same trend processing on the original data of the evaluation indicators. We use the reciprocal method to process the evaluation index with the same trend and obtain the extreme value consistency matrix. The calculation formula is as follows:

5 $$x_{i1}^ \ast = \frac{1}{{x_{i1}}}\, \left( {i = 1,2, \cdots ,m} \right)$$

Step 2: Compute for the standardized matrix.

Standardize the extremum consistency matrix based on the communalities of variables, calculate the standardized matrix *Z* = (*Z*_*ij*_). The calculation formula is as follows.

6 $$z_{ij} = \frac{{x_{ij}^ \ast }}{{\sqrt {\mathop {\sum}\limits_{i = 1}^m {x_{ij}^{ \ast 2}} } }}\, \left( {i = 1,2, \cdots ,m;j = 1,2, \cdots ,n} \right)$$

Step 3: Calculate the information entropy.

Measure the information entropy value *e*_*j*_ and the information utility value *d*_*j*_ of index *j*. The calculation formula is as follows:

7 $$e_j = - k\mathop {\sum}\limits_{i = 1}^m {y_{ij}\ln y_{ij}} ,\,{{{\mathrm{thereinto}}}}\,y_{ij} = \frac{{z_{ij}}}{{\mathop {\sum}\limits_{i = 1}^m {z_{ij}} }}\,\left( {0 \le y_{ij} \le 1} \right),\,k = \frac{1}{{\ln m}}$$

8 $$d_j = 1 - e_j$$

Step 4: Compute for the weight for each sub-dimension.

Calculate the weight of evaluation index *j*. The calculation formula is as follows:

9 $$w_j = \frac{{d_j}}{{\mathop {\sum}\limits_{j = 1}^n {d_j} }},\,{{{\mathrm{thereinto}}}}\,w_j \in \left[ {0,1} \right],\,\mathop {\sum}\limits_{j = 1}^n {w_j} = 1$$

Step 5: Attain the weighted decision matrix.

Calculate the weighting matrix according to the weight of the evaluation index. The calculation formula is as follows:

10 $$R = ( {r_{ij}} )_{m \times n},\,{{{\mathrm{thereinto}}}}\,r_{ij} = w_j \times z_{ij}\left( {i = 1,2,...,\,m;\,j = 1,2,...,\,n} \right)$$

Step 6: Compute for the ideal solution.

Determine the positive ideal solution $S_j^ +$, Determine the negative ideal solution $S_j^ -$. The calculation formula is as follows.

11 $${S_j^+} = \left\{ {s_1^ + ,s_2^ + , \cdots ,s_j^ + , \cdots ,s_n^ + } \right\},\,{{{\mathrm{thereinto}}}}\, {s_j^ +} = \max ( {r_{1j},r_{2j}, \cdots ,r_{kj}, \cdots ,r_{nj}} )$$

12 $${S_j^-} = \left\{ {s_1^ - ,s_2^ - , \cdots ,s_j^ - , \cdots s_n^ - } \right\},\,{{{\mathrm{thereinto}}}}\,{s_j^ -} \,{s_j^ -}= \min ( {r_{1j},r_{2j}, \cdots ,r_{kj}, \cdots ,r_{nj}} )$$

Step 7: Determine the separation distance of each public service from the best public service and worst public service using Eqs. (13) and (14).

Calculate the Euclidean distance between the evaluation object and the positive ideal solution $D_i^ +$, and calculate the Euclidean distance between the evaluation object and the negative ideal solution $D_i^ -$. The calculation formula is as follows:

13 $$D_i^ + = \sqrt {\mathop {\sum}\limits_{j = 1}^n {\left( {S_{ij}^ + - r_{ij}} \right)} ^2}$$

14 $$D_i^ - = \sqrt {\mathop {\sum}\limits_{j = 1}^n {\left( {r_{ij} - S_{ij}^ - } \right)} ^2}$$

Step 8: Measure the closeness of each public service to the best public service using Eq. (15).

Calculate the relative proximity between each evaluation object and the optimal solution *C*_*i*_. The calculation formula is as follows:

15 $$C_i = \frac{{D_i^ - }}{{D_i^ + + D_i^ - }},{{{\mathrm{thereinto}}}}\,C_i \in \left[ {0,1} \right]\quad \quad \quad \left( {i = 1,2, \cdots ,m} \right)$$

The closer *C*_*i*_ is to 1, the closer the evaluation object is to the optimal evaluation level.

### Data

The research sample we used is panel data from 31 provinces in China from 2014 to 2019, and the data is from China Statistical Yearbook 2015–2020.

#### The weights of indicators of public services

The weight calculation is the core of the evaluation of the public service level. For the process of weight calculation, we use the method of gradual accumulation to calculate the weight of each indicator. First of all, we use the entropy method to calculate the weight of each secondary index and get the secondary index weight matrix. Secondly, calculate the first level index according to the weight matrix of the second level index, and then use the entropy method to calculate the weight of the first level index. Finally, calculate the weight matrix of the first level indicators.

Since the weights of the primary and secondary indicators are not equal each year, in order to make the evaluation results more comparable, we use the arithmetic average method to average the indicator weights from 2014 to 2019 to obtain the indicator weights. The weights of indicators of public services from 2014 to 2019 are shown in Table 2.

**Table 2:** The weights of indicators of public services.

	w2019	w2018	w2017	w2016	w2015	w2014	mean
Public basic education	0.1955	0.1822	0.1436	0.1342	0.1764	0.2243	0.176
Medical and health care	0.1714	0.1445	0.1295	0.12	0.146	0.1167	0.138
Social security and employment services	0.1133	0.0997	0.1777	0.0888	0.0893	0.1266	0.1159
Public welfare infrastructure	0.1485	0.1742	0.1671	0.1107	0.136	0.0941	0.1385
Public ecological environment	0.1791	0.2324	0.2211	0.3756	0.2514	0.2554	0.2525
Public Cultural Services	0.1922	0.167	0.1608	0.1707	0.2007	0.1829	0.1791

#### Descriptive statistics

According to the public service indicator system in Table 1, the public service judgment matrix of 31 provinces in China is constructed according to Formula (10). Then, according to the weight matrix in Table 2, we calculated the public service scores of 31 provinces in China from 2014 to 2019 according to Eqs. (11)–(15). Table 3 shows the descriptive statistics of explanatory variables (public service level), explanatory variables, and control variables.

**Table 3:** Descriptive statistics.

Variable	Mean	Std. dev.	Min	Max
PS	0.1637	0.1735	0.0275	0.9456
CR	0.7264	0.0506	0.6259	0.8941
URCR	0.6912	0.0453	0.6016	0.7993
RUCR	0.8240	0.0980	0.6259	1.1616
GAPCR	0.4647	0.0585	0.2999	0.5923
IS	1.2374	0.6612	0.6325	5.0221
UR	57.2439	12.7549	23.7100	89.6000
PGDP	55588.6800	25643.5600	23151.0000	153095.0000

From Table 3, in general, the public service level of China’s 31 provinces is relatively low, with an average value of 0.1637, indicating a large regional gap.

The province with the highest public service score (0.9456) is 34.39 times the province with the lowest public service score (0.0275). From a macro perspective, the spatial distribution of public services in China is highly related to the spatial pattern of China’s economic development. China’s economic development presents a gradient distribution pattern, specifically, the economic development of the eastern region is higher than that of the central region, and the economic development of the central region is better than that of the western region. Influenced by the gradient distribution pattern of China’s economic development, China’s public service level is also characterized by high public service levels in the east and low public service level in the west (Langea et al., 2017).

## Empirical results

### Regression analysis

Regarding the explained variable, the consumption rate of rural residents is higher than that of urban residents. According to Engel’s Law, with the increase in income, the consumption rate of residents will decline, especially the proportion of food expenditure in total expenditure (Bateman and Balmford, 2018). In China, the income level of urban residents is significantly higher than that of rural residents, resulting in a lower consumption rate for urban residents than that of rural residents (Zhou and Shi, 2019).

In terms of control variables, there is a large gap in economic development between different regions in China. In terms of per capita GDP, the province with the highest GDP (153,095 yuan) is more than six times that of the province with the lowest GDP (23,151 yuan), showing a spatial pattern of high economic development in the east and low economic development in the central and western regions. The level of urbanization and industrial structure conforms to the distribution pattern of China’s economic development (Li et al., 2020; Hong et al., 2021).

Table 4 shows the empirical analysis results of the impact of public services on Chinese residents’ consumption. Firstly, we analyze the overall impact of public services on Chinese residents’ consumption (Column 1); secondly, we analyze the impact of public services on Chinese urban residents’ consumption level (Column 2); thirdly, we analyze the impact of public services on Chinese rural residents’ consumption level (Column 3); finally, the impact of public services on the consumption gap between urban and rural residents in China is analyzed (Column 4).

**Table 4:** Results of empirical analysis.

	(1)	(2)	(3)	(4)
	CR	URCR	RUCR	GAPCR
PS	0.0947***(0.0273)	0.1348*** (0.0234)	0.0445** (0.0545)	−0.1340***(0.0256)
IS	−0.0242***(0.0072)	−0.0213***(0.0062)	−0.0344** (0.0143)	−0.0240***(0.0067)
UR	0.0029***(0.0007)	0.0033***(0.0006)	0.0022 (0.0014)	0.0009 (0.0007)
GDP	0.1145*** (0.0126)	0.1263*** (0.0143)	0.0871*** (0.0079)	0.0392*** (0.0123)
Constant	0.6643***(0.0239)	0.5825***(0.0205)	0.8289***(0.0478)	0.4203*** (0.0224)
*R*^2^	0.6956	0.3448	0.8081	0.3770
*N*	186	186	186	186

** and *** indicate significance at 5% and 1%, respectively.

The impact of public services on residents’ consumption is mainly reflected in the following two aspects. First of all, the income effect, with the improvement of public service supply level, will reduce consumer spending in the field of social public service level, indirectly increase residents’ disposable income, and lead to the improvement of residents’ consumption level (Czyewski et al., 2021). Second, the substitution effect. With the improvement of the supply level of public services, the relative price of public services will decline. When the income level of residents and the prices of other consumer goods remain unchanged, residents will increase public service consumption, leading to a decline in the consumption level of residents (Gao et al., 2019; Yang and Sun, 2020). Therefore, from the perspective of income effect, public services have an expansion effect on residents’ consumption, which will improve residents’ consumption level. However, from the perspective of the substitution effect, public services have shrunk residents’ consumption and reduced residents’ consumption levels.

According to the empirical results, the supply of public services has a significant role in promoting the consumption of Chinese residents, indicating that the income effect of public services on Chinese residents is greater than the substitution effect. However, due to the different resource endowments and income levels of urban and rural residents, the impact of public services on the consumption of urban and rural residents in China is also different (Song, 2022).

The empirical results show that the promotion effect of public services on the consumption level of Chinese urban residents (0.1348) is greater than that of Chinese rural residents (0.0445). The empirical results show that when the significance level is 1%, the regression coefficient of public services to the consumption gap between urban and rural residents in China is −0.1340. However, because the consumption gap between urban and rural residents is a negative indicator, public services have a significant role in reducing the consumption gap between urban and rural residents. With the improvement of public services, the consumption gap between urban and rural residents in China has been narrowing.

As for the control variables, the industrial structure (IS) generally inhibits the consumption of urban and rural residents, but it helps to narrow the consumption gap between urban and rural residents. The reason is that this paper uses the ratio of the output value of the tertiary industry to the output value of the secondary industry to measure the level of regional industrial structure. According to the research of Zhang Wenwu (2010), through empirical research on economic data from 1978 to 2008, it is found that China’s consumption causal effect is obviously superior to the secondary industry of the tertiary industry. The secondary industry’s causal effect on consumption is 1.715, and the tertiary industry’s dominant effect on consumption is 0.192. Therefore, the industrial structure measured by this ratio has a restraining effect on the consumption of urban and rural residents. The upgrading of industrial structure affects the urban–rural consumption gap through three channels: factor allocation effect, output effect and consumption demonstration effect. On the one hand, upgrading the industrial structure can promote economic growth, which helps to increase the non-agricultural employment of rural residents, thus improving the income level of rural residents, thereby expanding farmers’ consumption expenditure and narrowing the urban–rural consumption gap (Zhou et al., 2020). On the other hand, the upgrading of the industrial structure has expanded the production capacity of enterprises and the scale of consumer goods production, enabling rural residents to buy more kinds of consumer goods, which has played a leading role in rural residents’ consumption and helped to narrow the urban–rural consumption gap. In addition, the upgrading of industrial structure helps to promote rural residents’ transfer of employment to cities, indirectly affects their consumption concepts and consumption patterns, and forms a consumption demonstration effect, which will help to narrow the urban–rural consumption gap.

The improvement of urbanization level (UR) has significantly promoted the consumption of urban and rural residents and narrowed the consumption gap between urban and rural residents. The reason is that the improvement of urbanization rate can effectively narrow the income gap between urban and rural residents, thus driving rural residents’ consumption. Urbanization is a process in which the population is constantly transferred from rural areas to cities, and forms agglomeration in geographical space (Lin et al., 2021). Urbanization can effectively promote the improvement of residents’ consumption level through agglomeration effect, economies of scale and external economic effects.

China’s economic growth (GDP) has significantly promoted the consumption of urban and rural residents and narrowed the consumption gap between urban and rural residents. With economic growth, the purchasing power of residents will increase, the consumption demand will expand, and the consumption field will also continue to expand, which will inevitably lead to the optimization and upgrading of the consumption structure. At the same time, economic growth will also promote technological progress. Technological innovation will lead to the adjustment of the industrial structure, and then new products and new consumption areas will emerge, which will further lead to changes in the consumption structure. With economic growth, residents’ income will increase. However, due to the influence of income gap and consumption preference, the consumption growth of different income groups will be different. Generally, the income growth of urban residents is more than that of rural areas, and the consumption preference of urban residents is stronger than that of rural residents. Therefore, economic growth may lead to the widening of the gap between urban and rural residents’ consumption levels. (Wang et al., 2020). Therefore, only reasonable income distribution can lead to reasonable and effective consumption and form a reasonable consumption structure.

## Conclusion and policy implications

### Conclusion

Improving residents’ welfare level is the fundamental purpose of public service provision, and residents’ consumption is the direct embodiment of residents’ welfare level. Therefore, this paper discusses the impact of public service supply on residents’ consumption and analyzes the difference in the impact of public service provision on urban and rural residents’ consumption. Based on the panel data of 31 provinces in China from 2014 to 2019, using the panel regression analysis method, we found that at the 1% significance level, the provision of public services has a significant role in promoting the consumption of Chinese residents, and the role of public services in promoting the consumption of Chinese urban residents is greater than that of rural residents. And improving public services will help narrow the consumption gap between China’s urban and rural residents.

Our research enriches the theories of Keynesian economics and development economics. Keynesian believes that insufficient demand will lead to overproduction and huge unemployment, and increasing effective demand will help promote economic development. From the perspective of public service, we found an effective way to solve the problem of insufficient demand, so this study enriched the theory of Keynesian economics. And the case study of China will help to provide a reference for the formulation of public service policies and economic policies of developing countries in the context of global economic recession. Developing countries can choose to improve public services, which will stimulate domestic consumption and promote economic development, thus enriching the theory of development economics. However, due to the complexity of the content of public services, different types of public services have different impacts on residents’ consumption. Therefore, in future research, we can distinguish the types of public services and analyze the impact of different types of public services on residents’ consumption levels.

### Policy implications

#### (1) Increase the expenditure of basic public services

The fiscal expenditure on basic public services is conducive to the rational distribution of social wealth. The government should increase the proportion of basic public service expenditure in national fiscal expenditure and improve the overall level of social welfare. According to the empirical results, the provision of basic public services plays a significant positive role in promoting Chinese residents’ consumption. Therefore, it is necessary to increase the government’s expenditure on basic public services to promote residents’ consumption.

Specifically, under the guidance of fair thinking, on the one hand, it is necessary to attach importance to promoting the fairness of residents’ consumption environmental fairness through the equal provision of basic public services. First, we must give full play to the multiplication effects brought by the financial policy of basic public services. Through government infrastructure investment, improvement of environmental investment, increased scientific and technological investment, etc., promote the balanced economic development between regional economies, and further drive regional investment and employment, improve the underdevelopment of underdevelopment Regional economic environment. The second is to invest in infrastructure investment and construction, including hardware service facilities such as hydropower, transportation, and networks, to improve investment and consumer environment in underdeveloped areas, guide residents to release consumer vitality, and improve consumption level.

On the other hand, we should pay attention to the fairness of the total amount of residents’ consumption. In underdeveloped areas, basic public services should be used as a substitute for private consumption. In this way, the income of residents in public consumption can be saved so as to be put into other private consumption areas and gradually realize fairness in total consumption. For example, if the government increases investment in culture, sports, and other facilities and services in underdeveloped areas and guarantees residents’ basic consumption of spiritual and cultural life, residents’ expenditure in this aspect can be saved and this part of income can be applied to other cultural and entertainment consumption, thus increasing the overall cultural consumption of residents in this area.

#### (2) Optimize the configuration of basic public services between urban and rural areas

According to empirical results, public services have a significant role in promoting the gap between urban and rural residents’ consumption, but the promotion effect of public services on the consumption of urban residents in China (0.1348) is greater than rural residents (0.0445). Therefore, in the process of urban and rural allocation of basic public services, the supply of basic public services is promoted to tilt towards rural areas, and the government’s public financial budget is put into the field of rural medical care, education, and social security related to residents’ daily life.

First, considering that rural residents are prone to return to poverty due to illness and lack of social security to return to poverty after losing their labor force, the overall level of rural medical subsidies should be increased. The government should improve the rural medical service system, provide a welfare medical supply, and reduce the burden on rural residents to see a doctor.

Secondly, increase the investment in rural education infrastructure to improve the unreasonable educational level between urban and rural areas. The investment in education can be generally divided into two aspects: on the one hand, the education of minors can be increased by increasing the years of compulsory education or increasing the amount of financial aid for poor students; Another aspect is the education of the labor force, which can be carried out by means of vocational and technical training.

Thirdly, improve the social security system. The government can increase the government expenditure on basic public services to establish a living security system that covers more people and guarantees more vigorously, and further improve the unemployment and old-age insurance system in rural areas, so that rural residents can enjoy unemployment insurance and old-age insurance similar to urban workers, so as to solve their worries.

## Data Availability

All data analyzed are contained in the paper.

## Footnotes

1 China Statistical Yearbook.

2 World Bank Data base.
